# Gut microbial diversity among Yorkshire, Landrace and Duroc boars and its impact on semen quality

**DOI:** 10.1186/s13568-022-01496-6

**Published:** 2022-12-23

**Authors:** Jiawei Li, Yuhang Li, Meixia Cheng, Fengchun Ye, Wen Li, Cong Wang, Yuxuan Huang, Yan Wu, Rui Xuan, Guanyuan Liu, Jianhua Huang

**Affiliations:** 1 College of Life Science, Jiangxi Science and Technology Normal University, Nanchang, China; 2 Laboratory X, Animal Husbandry and Veterinary Bureau of Yugan County, Shangrao, China; 3 Jiangxi Yifeng County Qiaoxi Veterinary Station, Yichun, China; 4 Changsheng Town People’s Government of Ningdu County, Ganzhou, China

**Keywords:** Purebred boars, Gut microbiota, Diversity, Semen performance, Age

## Abstract

The gut microbiota plays an important role in pig health and performance, particularly in host growth and fecundity. In present study, the characteristics and diversity of gut microbiota in fine purebred boars from three-way crossbred “Duroc×Landrace×Yorkshire” pigs were investigated using 16 S rRNA gene sequencing. The results showed that the three breeds of boars shared similar gut microbiota, yet there remain slight differences at the family/genus level. At the family level, *Ruminococcaceae*, *Streptococcaceae* and *Lactobacillaceae* have the highest abundance in Landrace, while *Rikenellaceae* and *f_p_251_o5* have the highest abundance in Duroc. The abundance of *Prevotellaceae*, *Lachnospiraceae* and *Spirochaetaceae* in intestinal of Yorkshire were higher than that of Landrace and Duroc. In addition, ten and six biomarkers were identified in the microbiota across breeds and months of age, respectively. Moreover, we evaluated the effect of gut microbiota on boar semen quality, revealing that Duroc had the strongest sperm vitality, significantly associated with the genus *Rikenellaceae_PC9_gut_group*. In addition, the spermatogenesis ability and sperm production improved gradually along with increase of age. In conclusion, this study provides a reference for understanding the gut microbiota composition of purebred boars used for three-way crosses and their impact on semen performance.

**Supplementary Information:**

The online version contains supplementary material available at 10.1186/s13568-022-01496-6.

## Introduction

Crossbreeding has been widely used in the pig industry as a very effective means to produce high-performance crossbred pigs that are more adaptable to complex environments than the original breeds and lines. The three-way crossbred pigs favored by pig farmers as their fast growth, strong resistance to adversity, high fecundity and significant economic benefits occupied a huge market share. The selection of parents is essential for the acquisition of excellent three-way crossbred pigs. The offspring of three-way crossbred of Yorkshire, Landrace and Duroc currently showed significant hybridization advantages. At first, the Landrace and Yorkshire with remarkable growth and development were selected as parents for crossbreeding to obtain binary crossbred pigs with superior traits inherited from both parents. Afterwards, the female parent from the binary crossbred pigs would be crossed with the well-grown Duroc boars to produce three-way crossbred pigs with excellent meat quality, fertility and adaptability(Lan et al. 2015; Xiao 2015).

The quality of boars is an important indicator in the pig industry, and highly productive boars contribute to increased progeny production and piglet viability. Duroc, an excellent paternal breed has strong adaptability, robustness, toughness, and strong resistance to stress, while their reproductive performance and high temperature endurance are relatively poor (Li et al. 2020). Both Yorkshire and Landrace have the advantages of good reproductive performance, fast growth and high feed conversion rate, so they are often used as hybrid parents (Fan et al. 2005). In addition, the lean meat rate of Duroc is significantly higher than that of Yorkshire and Landrace (Guo et al. 2019; Qu et al. 2021). The disease resistance and meat quality of the three-way crossbred pigs obtained after crossing could be fully reflected. In the current pig breeding industry, artificial insemination is a commonly used reproductive technology, which means that the fertility of the boar is particularly important for the reproduction of the offspring. Therefore, studies of boar semen quality are of great economic value for the pig breeding industry (Smital 2009). The quality of boar semen is significantly better in autumn and winter than in spring and summer, while its production declines rapidly after 36 months of age. (Chen et al. 2022; Shang et al. 2020). It was found that different breeds, seasons and ages would affect the semen quality of boars.

Gut microbes have a crucial role in maintaining a variety of physiological activities and health of the host. Numerous studies have shown that composition of gut microbiota in pigs affects their growth, metabolism, reproduction and even meat quality. It has important theoretical and practical significance to improve the production performance by promoting the intestinal microbiota environment (Swords et al. 1993; Yang et al. 2014). Studies have found that the supplement of astragalus to feed improved the intestinal microbiota thereby reducing morbidity in three-way crossbred pigs, while increasing the daily weight gain of three-breed hybrid pigs and improving feed conversion (Mi et al. 2021; Zhang et al. 2021). Wang et al.( 2020) found that heat stress disrupts the balance between beneficial and harmful flora in the intestine, resulting in a disturbance of microbiota. A study pointed out that the feed intake efficiency of Duroc, Landrace, and Yorkshire may be mainly reflected in the compositional differences of gut microbes (Bergamaschi et al. 2020). In addition, the lack of vitamins and other substances produced by intestinal microorganisms is likely to affect the quality of boar semen and lead to infertility of sows, as well as slow fattening and sexual maturity, which seriously affects the development of pig breeding (Lin et al. 2002; Liu et al. 2011).

At present, there are few reports on the influence of gut microbes on the production of these three stock boars. In this study, we used 16 S rRNA gene sequencing to analyze the composition and structure of the intestinal microbial communities of Yorkshire, Landrace and Duroc boars in the same feeding conditions, aiming to reveal the differences in the composition and diversity of the flora among them. More importantly, it is the ability to understand the differences between the intestinal microbiota of different boars and their effects on breeding, as well as the possible effects on the diversity and metabolic function of the intestinal microbiota of the species.

## Materials and methods

### Animals samples

All animals in this study were obtained from a large standardized breeding farm in Jiangxi Province. Fecal samples were collected from 26 healthy purebred boars, including 8 Duroc (D), 9 Landrace (L) and 9 Yorkshire(Y), all of which were excellent male parent for three-way crossbred. Before sampling, all individuals were provided with the same growth environment and diet. The ambient temperature in winter was not lower than 16 °C, and not higher than 28 °C in summer, with humidity controlled at 65% or less. The diet was fed twice a day (morning 10:00 and 4:00 pm), with the feeding amount will be slightly adjusted according to the body condition of the pigs. The entire sampling process was strictly carried out in accordance with standard procedures. The sampling equipment was always sterilized with 75% ethanol before operation. To reduce the risk of cross-contamination, each individual was sampled separately. The fresh fecal samples were placed in pre-prepared sterile cryovials and then stored in a liquid nitrogen container. After all sampling was completed, brought them back to the laboratory and placed it in a − 80 °C ultra-low temperature freezer for freezing until DNA extraction. The sperm indexes in this study were determined by professional staff in breeding farms.

### Genomic DNA extraction and PCR amplification

Extraction of total fecal DNA was performed using the TGuide S96 Magnetic Bead Soil/Feces Genomic DNA Extraction Kit following the manufacturer’s instructions. The concentration and integrity of the extracted DNA were assessed by microplate reader and 1.8% agarose gel electrophoresis. After obtaining qualified DNA samples, specific primers (338F: 5′-ACTCCTACGGGAGGCAGCA-3′ and 806R: 5′-GGACTACHVGGGTWTCTAAT-3′) were used to amplify the V3-V4 hypervariable region of bacterial 16 S rRNA, where the primers were designed according to the sequence conserved region. And then sequencing adapters were added to the ends of the primers for PCR amplification. The recovered PCR products were purified, quantified and homogenized to generate a sequencing library. The constructed libraries were tested to quality inspection by the Qsep-400 method. The qualified libraries were paired-end sequencing using Illumina Novaseq 6000.

### Sequencing processing and taxonomic analysis

The raw data obtained after sequencing needs to be screened and preprocessed according to the sequence quality to satisfy the subsequent analysis. First, Trimmomatic (Bolger et al. 2014) (Version 0.33) was employed to perform quality filtering on the original data with an average quality score less than 20. Cutadapt (Martin 2011) (Version 1.9.1) was used to identify and remove primer sequences to generate clean reads. Then USEARCH (Edgar 2013) (Version 10) was applied to splice the paired-end reads with overlapping area greater than 10 bp. The chimeric sequences were checked and removed by UCHIME (Edgar et al. 2011) (Version 8.1). Finally, high-quality effective sequences were obtained for subsequent analysis. The valid sequences were clustered with 97% similarity using UPARSE (Edgar 2010) to obtain consistent operational taxonomic units (OTUs). The OTUs sequences were taxonomically annotated using Naive Bayes classifier (NBC) based on SILVA reference database to obtain taxonomic information on the species corresponding to each OUT, and then the community composition of each sample was counted at each taxonomic level.

### **Data analysis**

Alpha diversity analysis was used to measure the species abundance and diversity within the group. The richness and diversity of the samples were evaluated by using the QIIME2 (Bolyen et al. 2019) software to count the ACE index and Shannon index of each group. Sample rarefaction curves and Shannon diversity index curves were used to determine whether the amount of sequencing data was sufficient to reflect species diversity and richness in the samples. Beta diversity can evaluate the differential distribution of species community composition between groups. At the same time, principal coordinate analysis (PCoA) and non-metric multidimensional scale (NMDS) were used to explain the differences of microbial community composition based on distance matrix.

The linear discriminant analysis effect size (LEfSe) (Segata et al. 2011) was performed to identify specific biomarkers between groups with LDA scores > 4 at multiple taxonomic levels. The relationship between the flora and various environmental factors was investigated by RDA/CCA analysis. Functional prediction was performed using PICRUSt2 (Douglas et al. 2019) to analyze functional differences between different samples or subgroups, in which IMG microbial genome data was used to output functional information and infer the functional gene composition in the samples. KEGG metabolic pathways were analyzed to determine the differences and changes in the metabolic pathways of functional genes in microbial communities between groups. Analysis of variance (ANOVA) combined with pairwise T-test were employed to analyze the inter group differences for results and P < 0.05 was considered statistically significant.

## Results

### Characteristic of semen quality along boar breeds and age

Sperm quality plays an important role in the fertility rate, meat quality, stress resistance and other characteristics of three-way crossbred pigs. Therefore, we investigated the influence of breed and age on sperm quality. The results showed that the sperm density and vitality were visibly lowest in Yorkshire, while the semen volume was no significant difference among the three breeds. In addition, the sperm density was notably higher in Duroc than that of the other two breeds, and the sperm motility of Landrace was significantly the highest. The analysis of the influence of different days of age on sperm quality showed that semen volume and sperm density were significantly increased and maintained stable with the increase of days, but there was no obvious difference in sperm motility between different age groups (Additional file 1: Table. S1).

### Overview of the gut microbiota of three boars

A total of 2,080,071 original reads were obtained from 26 boar samples using bacterial 16 S rRNA gene sequencing. After removing low-quality and chimeric sequences, 2,016,026 valid reads were obtained, with an average of 77,544 reads per breed, which were employed for subsequent data analysis. The average length of reads is about 416.69 bp, the GC content is 53.30%, and the overall Q20 > 99%. The effective sequences of all samples were clustered into 810 OTUs, of which 793 were shared among the three group (Additional file 1: Fig. S1), implying high sequence similarity between the gut microbiota of these three breeds of pigs. Species abundance was then estimated using the ACE index, and bacterial diversity was estimated using the Shannon index. The results showed that Yorkshire had the highest ACE index. Their gut microbial community richness significantly higher than that of the Duroc and Landrace (p < 0.05), but the Shannon index showed no significant difference in the bacterial community diversity among the three breeds. We explored the effect on diversity of gut microbes along the increase of age in three pig breeds. The result showed that the community richness of gut microbes in pigs was notably improved with the increase of time, while the community diversity with no significantly change (Additional file 1: Fig. S3). In addition, the average coverage of 99.91% indicates that most of the microorganisms in the feces have been captured (Table 1). To determine whether the sequencing depth was sufficient to provide an overview of the gut microbiota, rarefaction curves of OTU numbers for each individual were generated as well as Shannon curves (Additional file 1: Fig. S2), both of which eventually flattened. This indicates that there are sufficient reads to represent each microbiome to ensure adequacy and accuracy of subsequent analysis.

**Table 1:** Summary statistics of different breeds in this study

Group	Age composition (number)	Effective reads	OTUs	ACE	Shannon	Coverage (%)
Duroc	N(1), F(6), T(1)	77,672	798	720.6677$\pm$5.2038^a^	6.8936$\pm$0.1610	99.91
Landrace	N(1), F(7), T(1)	77,002	800	722.7039$\pm$5.5286^b^	7.0103$\pm$0.1686	99.91
Yorkshire	N(6), F(1), T(2)	77,959	810	743.7708$\pm$8.0854^a,b^	7.1458$\pm$0.1423	99.92

Note: N, F and T represent ages of nine, fifteen and twenty months respectively. The same letters in the superscript indicated a significant difference between the two groups (p < 0.05)

### Composition and comparison of gut microbial communities of three boars

**Fig. 1:**
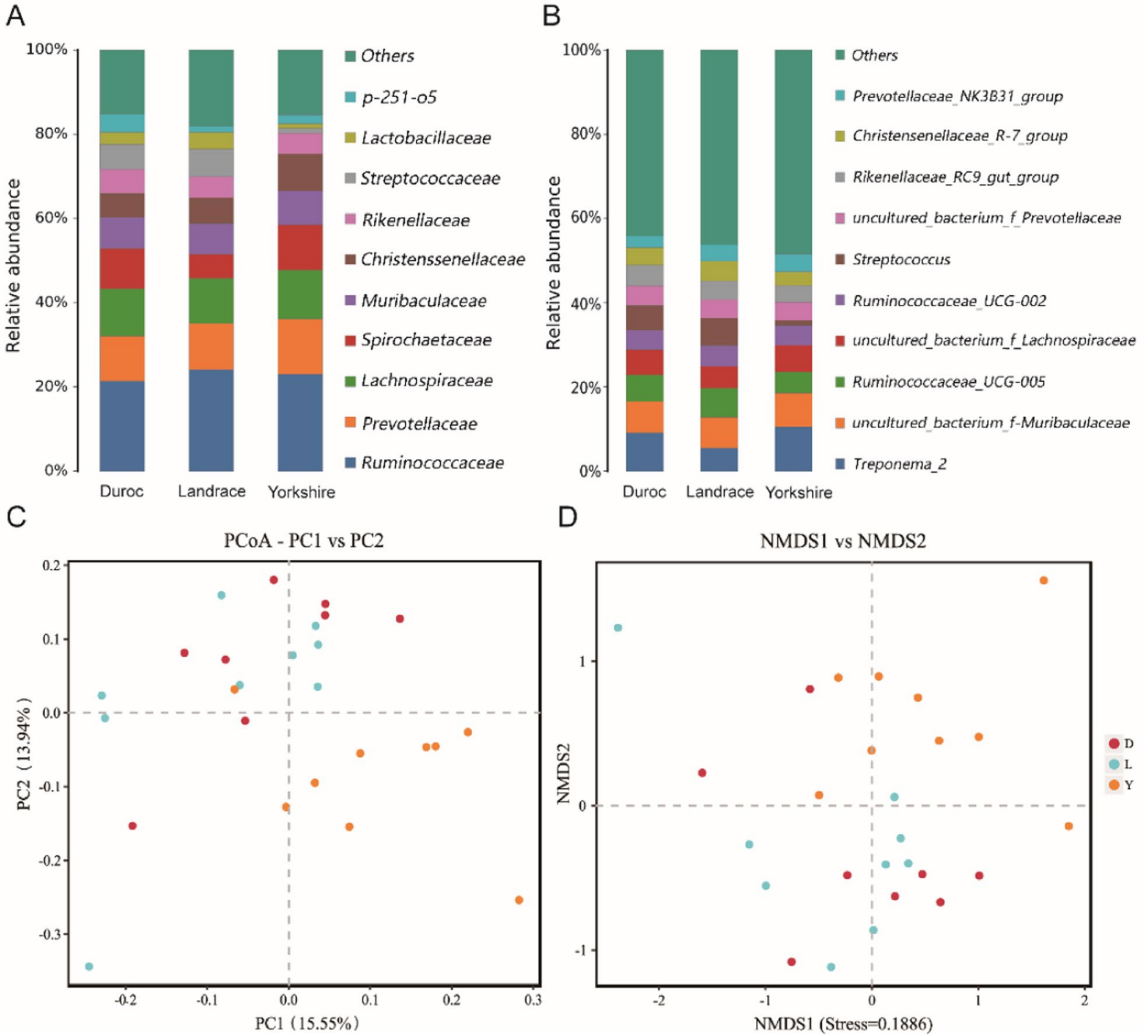
**A** Histogram of species abundance at the family level of gut microbiota of different breeds; **B** Histogram of species abundance at the genus level of gut microbiota of different breeds; **C** PCoA of three breed samples; **D** Nonmetric Multidimensional Scaling (NMDS) of bacterial communities of three breeds of pigs (Duroc, Landrace and Yorkshire) based on Bray-Curtis distance

The abundance of gut microbiota in all boars showed that *Firmicutes* (55.29%), *Bacteroidetes* (30.93%) and *Spirochaetes* (8.62%) were the dominant phyla, accounting for more than 90% of all sequences. This is basically consistent with the previous report on the composition of the gut microbiota in pigs (Tan et al. 2018). To further analyze the differences of gut microbiota among different breeds, we found that the top five dominant bacterial families in boar were *Ruminococcaceae*, *Prevotellaceae*, *Lachnospiraceae*, *Spirochaetaceae* and *Muribaculaceae*. Specifically, *Ruminococcaceae* accounted for 24.16% in Landrace, 23.04% in Yorkshire, and 21.40% in Duroc. *Ruminococcaceae* had the highest abundance in Landrace. Concurrently, the abundance of *Streptococcaceae* (6.53%) and *Lactobacillaceae* (3.97%) in Landrace were higher than the other two breeds. *Rikenellaceae* (5.76%) and f_p_251_o5(4.34%) had the highest abundant in Duroc. The abundances of *Prevotellaceae* (13.9%), *Lachnospiraceae* (11.61%), *Spirochnospiraceae* (10.78%), *Muribaculaceae* (8.04%) and *Christensenellaceae* (8.77%) in Yorkshire were higher than those of the other two species. The proportion of *Streptococcaceae* was 1.18%, which was the lowest in Yorkshire pigs (Fig. 1A). At top 10 core genera, the relative abundance of *Treponema_2* in Landrace was significantly lower than that in other two breeds, *Streptococcus* and *Ruminococcaceae_UCG_005* in Yorkshire were much lower, *Prevotellaceae_NK3B31_group* was the lowest in Duroc, while the other core genera were relatively closed (Fig. 1B)

PCoA was performed to demonstrate the diversity of samples or groups. It was observed that there was no obvious separation among the samples as a whole but there were still slight differences among different breeds. The ANOSIM analysis (R = 0.219, p < 0.001) further confirmed the significant differences in bacterial community structure among different breeds (Additional file 1: Fig. S4A). Intestinal microbiota samples were clustered according to pig breeds, and the intestinal microbiota of approximately Yorkshire had greater variation and were significantly different from other pig breeds. In contrast, Landrace and Duroc had more similar and stable gut microbial communities. (Fig. 1C). We explored the effect on diversity of gut microbes along the increase of age in three pig breeds. The result showed that the community richness of gut microbes in pigs was notably improved with the increase of time, while the community diversity with no significantly change (Fig. 1D, Additional file 1: Fig. S4B).

### Biomarker identification and functional prediction of pigs

**Fig. 2:**
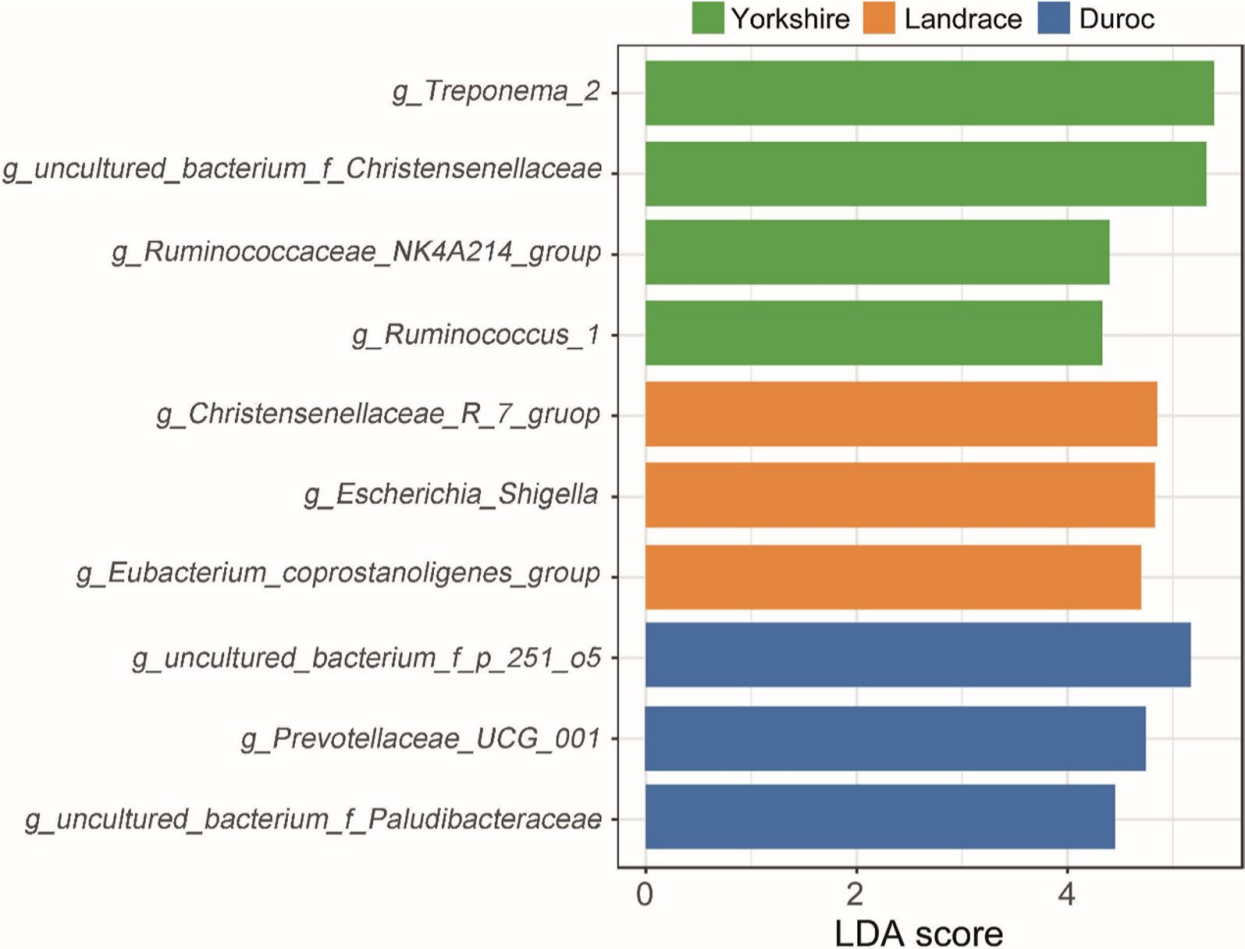
Biomarkers identified at the genus level by LEfSe analysis

Differential taxonomic features at the genus level were identified by LEfSe analysis as biomarkers for each purebred boar in Yorkshire, Landrace and Duroc. A total of 10 taxa with significant differences in relative abundance were identified by LDA score > 4 (Fig. 2). Yorkshire have been identified in four genera, including *Treponema_2*, *Christensenellaceae*, *Ruminococcaceae_NK4A214_group* and *Ruminococcus_1*. Three microbial genera were identified as biomarkers in the intestine of Yorkshire and Landrace, respectively. The main biomarker for Landrace was *Christensenellaceae_R_7_group*, while *f_p_215_o5* and *Prevotellaceae_UCG_004* were mainly found in Duroc

.

Age is an important factor affecting the intestinal microbiota of pigs. The abundance of intestinal flora may change significantly even in the same breed of pigs at different stages of growth and development (Qu et al. 2021). We further compared the effect of month age on biomarkers in the gut microbiota and identified 6 taxa with significant differences in relative abundance using the same criteria (Additional file 1: Fig. S5). The result showed that *Prevotella_9* was mainly found in nine-month-old pigs. *Turicibacter* was mainly found in fifteen-month-old pigs. *Ruminococcaceae_UGC_013* was mainly existed in twenty-month-old pigs. This further confirms that month age was a significant effect on the gut microflora.

In addition, in order to better understand the functional of gut microbiota, PICRUSt2 (Douglas et al. 2019) was applied to investigate functional differences of gut microbiota in different boar breeds. The function prediction of KEGG homolog (KOs) was performed using 16 S rDNA gene sequences. We found 46 functional categories in functional prediction, and 8 functional categories were significantly different across breeds (Additional file 1: Table S1). The results showed that the biosynthesis of secondary metabolites, transport and catabolism in Duroc were significantly higher than that in Landrace. These metabolites play important roles in many biological processes. Lipid metabolism, other amino acid metabolism and nervous system function in Landrace were significantly higher than that in other two breeds. The biosynthesis of secondary metabolites, cell growth and death, and environmental adaptation of Yorkshire were significantly enriched than that in Landrace, which may be related to the physical fitness and adaptability of Yorkshire higher than those of Landrace.

### Effects of boar gut microbiota on semen quality

**Fig. 3:**
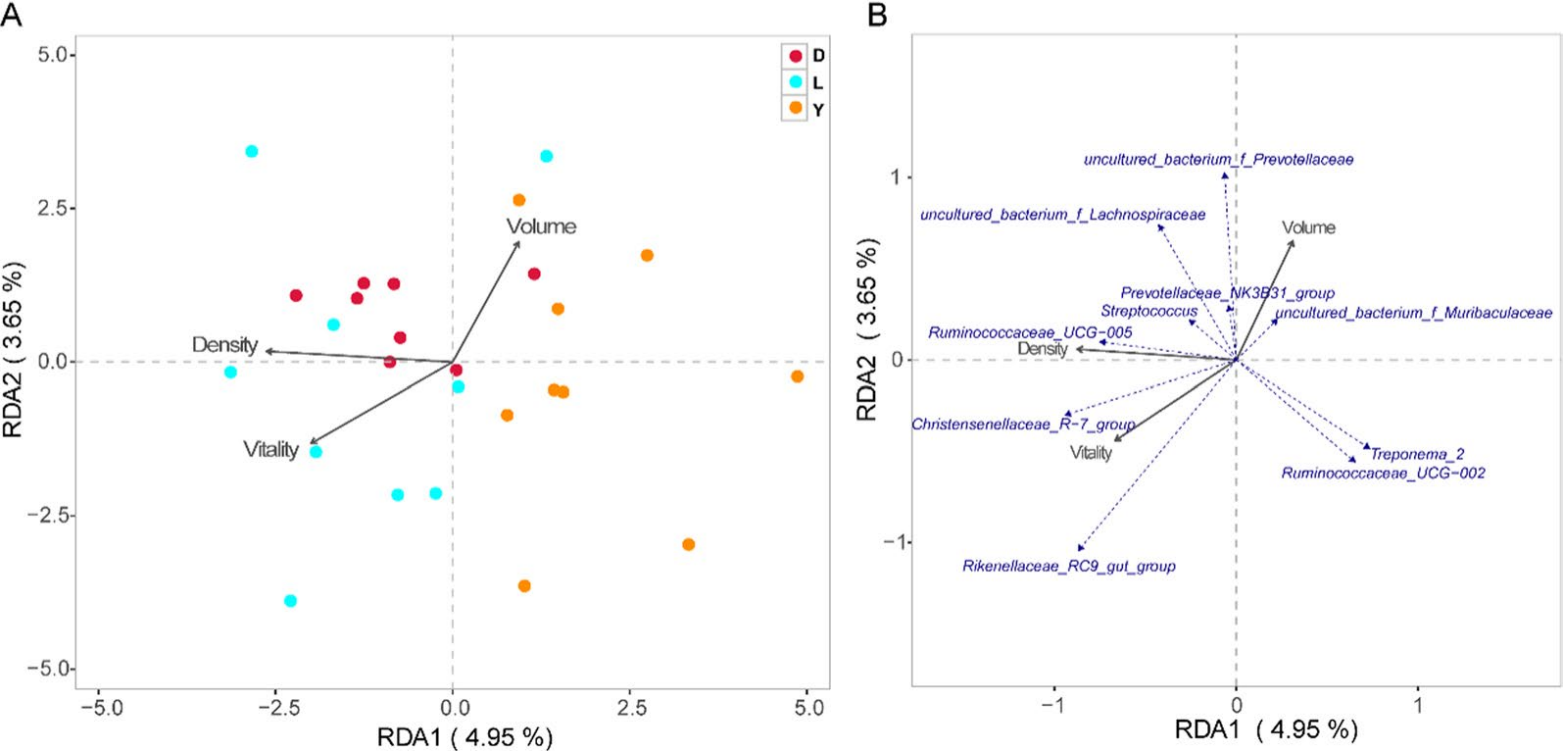
**A** Correlation analysis between three varieties and environmental factors; **B** Correlation analysis between the top ten species of genus level abundance and environmental factors. The scale on the horizontal and vertical coordinates are the values generated by each sample or species in the regression analysis calculation with environmental factors; points represent samples; the relationship between element points and points in the figure is represented by distance, the closer the distance is, the sample is represented composition is similar; the relationship between rays and rays is represented by an included angle. An obtuse angle indicates a negative correlation between the two physiological indicators, and an acute angle represents a positive correlation. The smaller the angle, the higher the correlation. The longer the arrow, the greater the influence on the sample distribution, and the gray arrows represent different environmental factors

To explore the relationship between the intestinal microbiota and semen quality of boar breeds, RDA analysis was conducted based on microbial environmental factors. The results showed that Yorkshire had the strongest correlation with semen volume, Duroc had the strongest correlation with sperm density, Duroc and Landrace had higher correlation with total sperm vitality than that of Yorkshire (Fig. 3 A). Simultaneously, through the correlation analysis of the top ten bacterial genera with environmental factors, it was found that *uncultured_bacterium_f_prevotellaceae* had the highest correlation with semen volume. *Ruminococcaceaae_UCG_005* had the highest correlation with sperm density. *Rikenellaceae_RC9_gut_group* had a positive and highest correlation with total sperm vitality (Fig. 3B).

In order to further explore the relationship between different ages and pig gut microbes, samples of different ages were also analyzed for microbial environmental factors. The 20 months of pigs was the strongest associated with sperm collection, while 15 months of pigs was positive connected with sperm density (Additional file 1: Fig. S6).

## Discussion

The intestines of poultry are populated by thousands of microbes, which are modified by many factors, such as host, time (growth stage), space (different intestinal segments), and feeding conditions. In the pig breeding industry, people tend to raise varieties with high production performance, fast growth and high feed conversion rate. Therefore, the three-way crossbred pigs of Landrace, Yorkshire and Duroc have been widely bred. This study aimed to explore the differences of gut microbiota of three purebred boar breeds (Landrace, Yorkshire, and Duroc) and their impact on cross-breeding.

At present, studies have shown that environment, breed, and age could affect the gut microbes of animals (Qu et al. 2021; Sender et al. 2016). We found 5 unique OTUs in Yorkshire, representing *Rikenellaceae*, *Saccharimonadaceae*, *Erysipelotrichaceae*, *Burkholderiaceae* and *Prevotellaceae*. It has been proved that *Prevotellaceae* in the intestines of pigs fed with feed formula could activate its metabolites through specific signaling pathways to cause chronic inflammatory response in the host and significantly increase fat deposition in the host. Moreover, *Prevotella* involved in polysaccharide degradation and amino acid metabolism in the host could affect fat production and hepatic glycogen storage in pigs (Chen et al. 2021; Fang et al. 2017). The lowest percentage of lean meat in Yorkshire may be related to *Prevotellaceae*. Analysis of the association between gut microbes and age showed that the *Rikenellaceae*, *Burkholderiaceae* and *Prevotellaceae* were unique OTUs in 9-month-old pigs, which may be to related to the fact that pigs at rapid growth stage required a lot of energy. After further microbial diversity analysis, we found that the gut microbiota were clustered according to their breeds and ages. It was determined that the gut microbiota between pigs of different breeds and ages was different but not significant. This probably related to the same growing environment and feeding environment in which the samples were collected. It has been reported that the proportion of *Firmicutes* in the intestinal tract of obese pigs is higher, while the proportion of *Bacteroidetes* is lower, and the opposite is true for lean meat types (Swords et al. 1993). With the increase of age, the abundance of *Firmicutes* and *Bacteroidetes* increased (Zhao et al. 2015). In this study, the abundance of *Firmicutes* in the intestines of Landrace was the highest, but its lean meat rate was in the middle level of the three breeds, which may be due to the lower average age of Landrace.

Javurek et al. (Javurek et al. 2017) found that the gut microbiota of mice fed a high-fat diet changed and affected the semen quality. A study using gut microbes to analyze boar semen utilization found that it was negatively correlated with the abundance of *Ruminococcus* and *Sphingobium* (Guo et al. 2020). This may be one of the reasons why Duroc is an excellent male breed. Thus, it is further speculated that the high sperm quality of Duroc may be related to the low abundance of *Ruminococcaceae*. Ding et al. ( 2020) found that the higher the abundance of *Bacteroides* and *Prevotella*, the lower the sperm vitality of the host. High abundance of Bacteroides and Prevotella negatively correlated with sperm quality in Yorkshire gut. could be one of the key factors. Our results are similar to previous studies provided a compelling support that Yorkshire is used as the female parent in ternary pigs. At the same time, study has proved that the semen collection volume of Duroc is less than that of Landrace and Yorkshire, while the difference between Landrace and Yorkshire is not significant (Zhu et al. 2016). In this study, the correlation between Yorkshire and sperm collection is the strongest, which is consistent with its results. Fraser et al.( 2016) showed that after sexual maturity, the semen volume showed an upward trend with the increase of age, which was consistent with the results of the strongest correlation between the 20-month-old Duroc. With increasing age, the production capacity of boar sperm continues to increase. Ren et al. ( 2007) found that the sperm vitality of Duroc, Yorkshire and Landrace decreased in turn. This is consistent with our findings. In addition, it was found in our study that the sperm density also decreased in the three breeds successively. *Ruminococcaceaae_UCG_002* was negatively correlated with total sperm motility. It has been demonstrated that *Ruminococcus* and *Sphingobium* abundances were negatively correlated with sperm utilization (Guo et al. 2020), leading to speculation that the abundance of *Ruminococcaceaae_UCG_002* may affect sperm vitality. Meanwhile, we found that *Rikenellaceae_PC9_gut_group* was positively and most strongly correlated with total sperm vitality. The highest abundance of *Rikenellaceae* was found in Duroc, leading to the hypothesis that it may be positively correlated with sperm vitality.

Although there were no significant differences in gut microbes among the three breeds, they were still be distinguished by biomarkers, which may explain the differences among breeds. *Prevotellaceae_UCG_001* is a biomarker in Duroc. The higher abundance of *Prevotellaceae* was negatively linked with the percentage of fat found in the weight loss experiment. Song et al. found that *Prevotellaceae_UCG_001* has low abundance in obese mice, and *Prevotellaceae* can degrade mucin and promote protein absorptionwhich may be related to the high lean meat rate of Duroc (Chen et al. 2013; Christensen et al. 2019; Song et al. 2019). *Prevotellaceae* is also an opportunistic pathogen caused intestinal inflammation, rheumatoid arthritis, bacterial vaginitis and other disease may be related to the poor adaptability to high temperature and low fertility of Duroc (Chen et al. 2021). Moreover, the *Christensenellaceae* as in Landrace is widespread in the animal gut and mucosa, which was significantly negatively correlated with metabolic diseases such as inflammation, fat deposition and metabolic syndrome. This may be related to the relatively low lean rate of Yorkshire (Waters Ley 2019). *Escherichia_Shigella* is a biomarker of Landrace. Study has shown that *Escherchia coli* is a pathogenic bacterium, which significantly increases in proportion and decreases in short-chain fatty acid content when piglets lose weight. This may be related to the weak physique, poor stress resistance and poor adaptability of Landrace (Sokol et al. 2017).

In conclusions, we investigated the characterize of gut microbiota in boar breeds using 16 S rRNA sequencing technology. We found that Duroc and Landrace as excellent hybrid males have high quality sperm, which response to breeds and age. Notably, the composition and function of gut microbiota in pigs were breed and age specific. Additionally, sperm quality of boars was positively correlated with the abundance of *Rikenellaceae*, while *Ruminococcaceaae_UCG-002* was oppositely. This study provided a valuable insight for understanding the effects of intestinal flora on the reproductive performance of boars.

## Supplementary Information

**Additional file 1: Figure S1.** Venn diagram of gut microbes in different varieties. **Figure S2.** Rarefaction curves for all samples;B Shannon curve for all samples.The abscissa is the number of randomly selectedsequencing strips, and the ordinate is the number of OTUs obtained based on thenumber of sequencing strips. Each curve represents a sample and is marked withdifferent colors. **Figure S3. **Comparison of alpha diversity metrics (ACE index and Shannon index) of different age at the OTU level.One-way ANOVA was used to compare the differences, andp < 0.05 was considered statistically significant.** Figure S4. **A Box plot indicating differences between different breeds by ANOSIM; B Box plot indicating differences between different ages by ANOSIM.** Figure S5.** Biomarkers identified at the genus level by LEfSe analysis of different ages. **Fig. S6.** Association analysis of different ages and environmental factors. **Table S1.** Semen quality of breeds and age. **Table S2.** Predicted functional differences in the KEGG pathways.

## Data Availability

The raw sequence data used in this manuscript have been deposited in the Genome Sequence Archive in National Genomics Data Center under accession number PRJCA009589.
