# Supplementary material for: Gut microbial diversity among Yorkshire, Landrace and Duroc boars and its impact on semen quality
Source: AMB Express. 2022 Dec 23;12:158. doi: 10.1186/s13568-022-01496-6 (PMC9789229; doi:10.1186/s13568-022-01496-6)
Supplement: Supplementary file 1 — Additional file 1: Figure S1. Venn diagram of gut microbes in different varieties. Figure S2. Rarefaction curves for all samples;B Shannon curve for all samples.The abscissa is the number of randomly selectedsequencing strips, and the ordinate is the number of OTUs obtained based on thenumber of sequencing strips. Each curve represents a sample and is marked withdifferent colors. Figure S3. Comparison of alpha diversity metrics (ACE index and Shannon index) of different age at the OTU level.One-way ANOVA was used to compare the differences, andp < 0.05 was considered statistically significant. Figure S4. A Box plot indicating differences between different breeds by ANOSIM; B Box plot indicating differences between different ages by ANOSIM. Figure S5. Biomarkers identified at the genus level by LEfSe analysis of different ages. Fig. S6. Association analysis of different ages and environmental factors. Table S1. Semen quality of breeds and age. Table S2. Predicted functional differences in the KEGG pathways. [file 13568_2022_1496_MOESM1_ESM.docx]

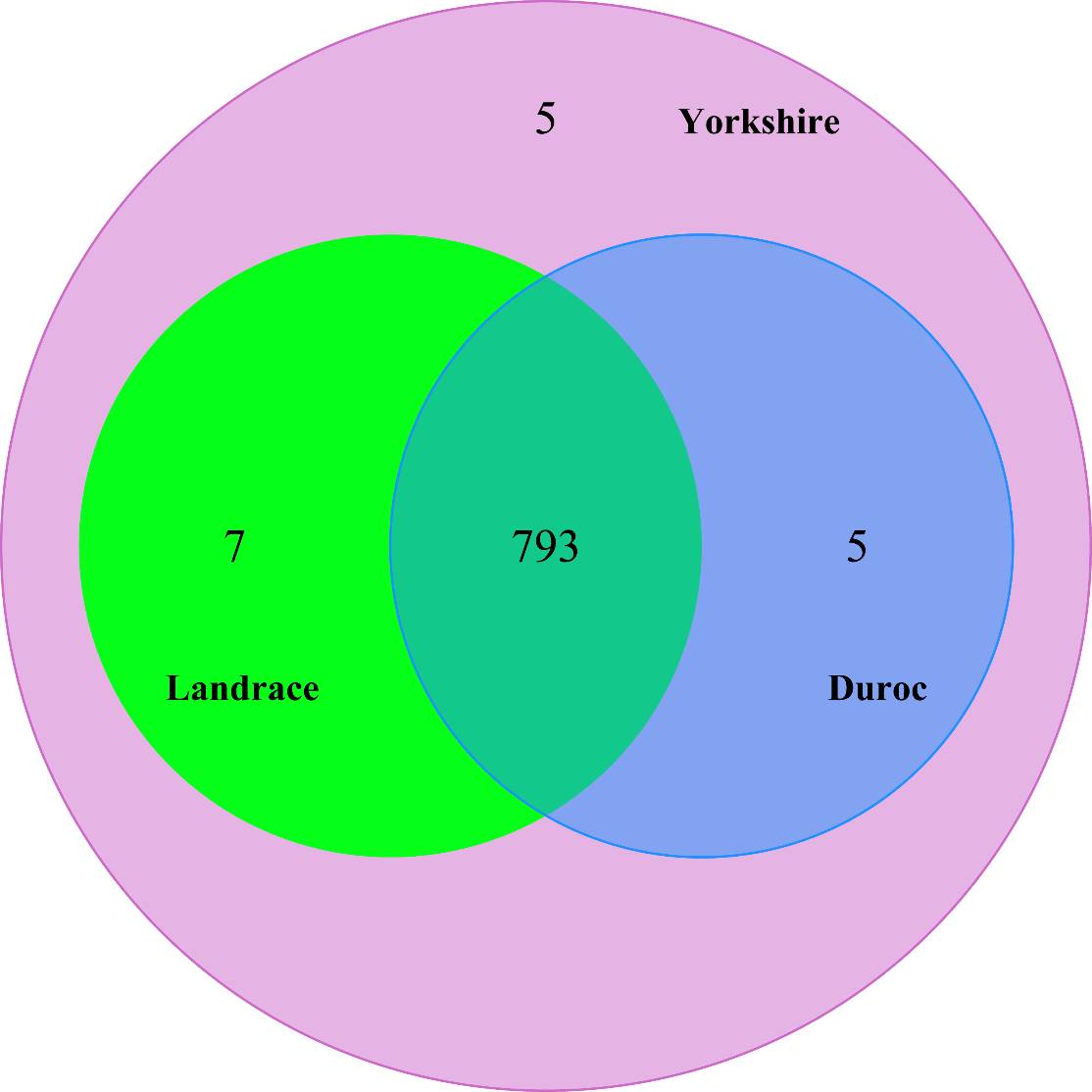


**Fig. S1.** Venn diagram of gut microbes in different varieties


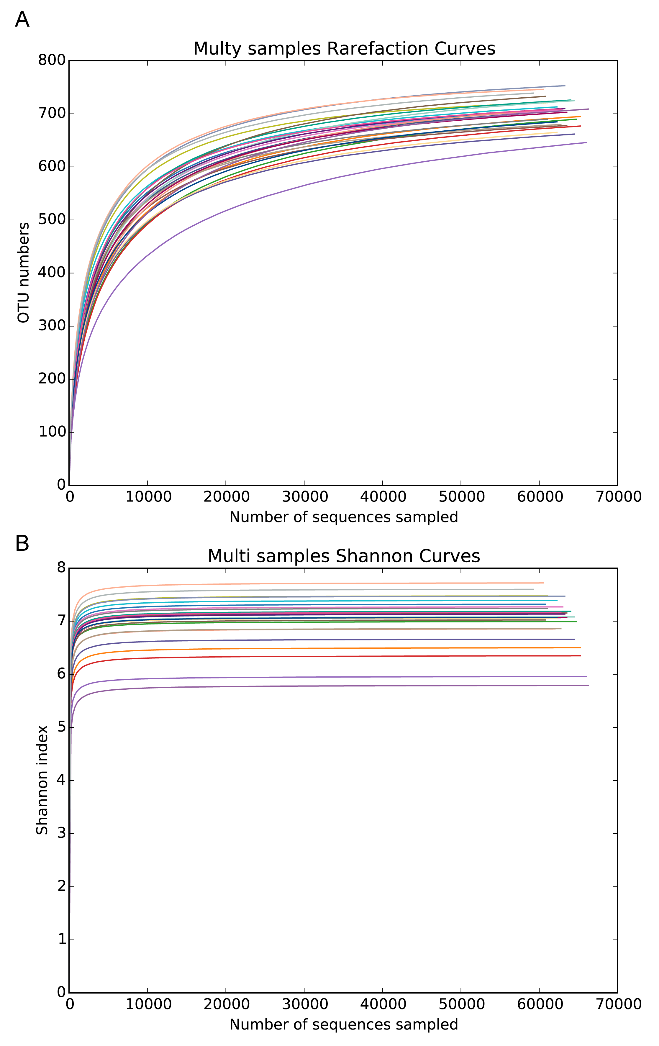


**Fig. S2.** A Rarefaction curves for all samples; B Shannon curve for all samples

Note: The abscissa is the number of randomly selected sequencing strips, and the ordinate is the number of OTUs obtained based on the number of sequencing strips. Each curve represents a sample and is marked with different colors.


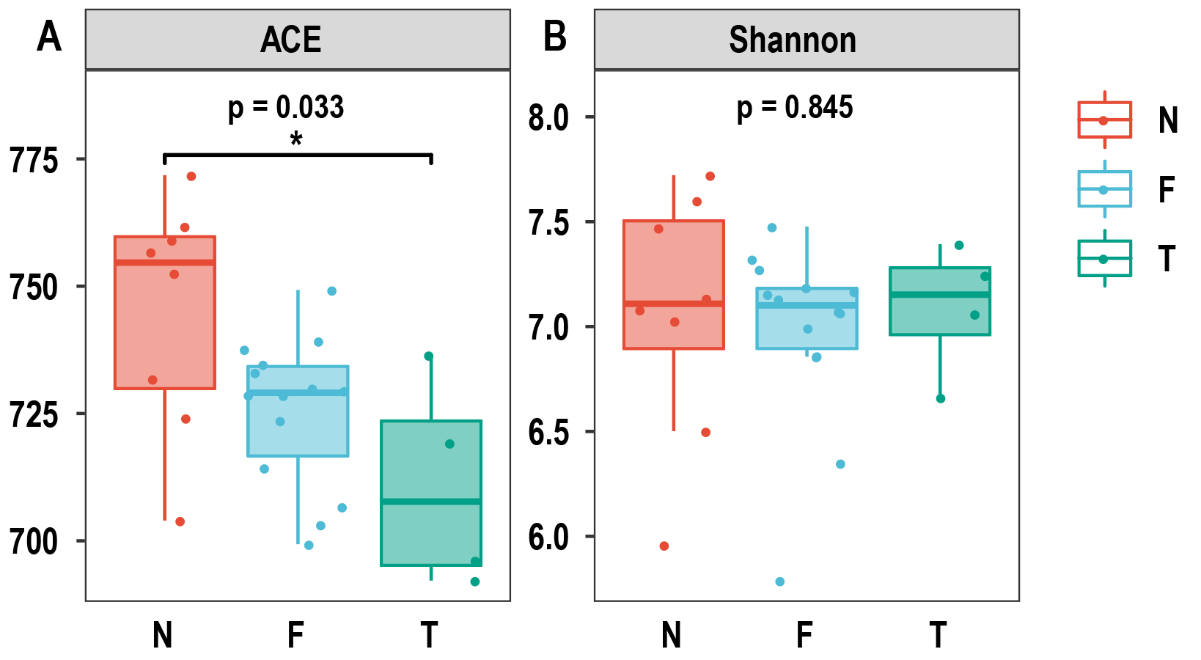


**Fig. S3.** Comparison of alpha diversity metrics (ACE index and Shannon index) of different age at the OTU level.

Note: One-way ANOVA was used to compare the differences, and p < 0.05 was considered statistically significant.


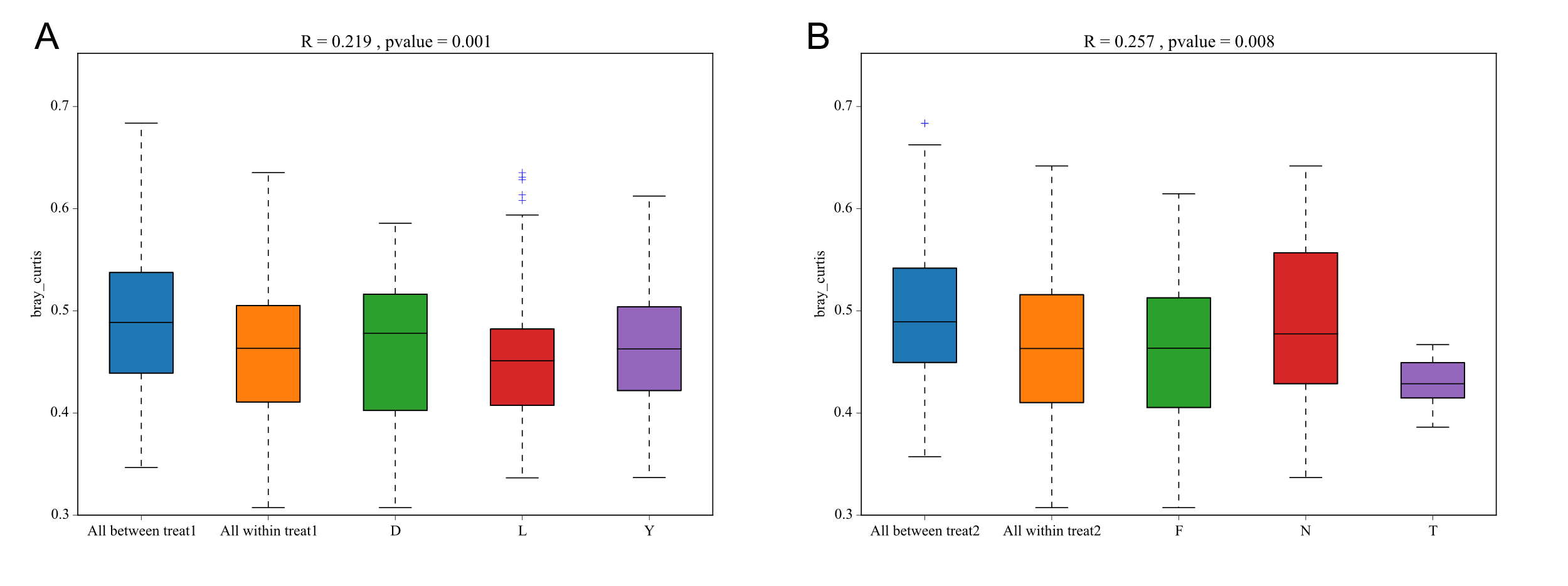


**Fig. S4.** A Box plot indicating differences between different breeds by ANOSIM; B Box plot indicating differences between different ages by ANOSIM


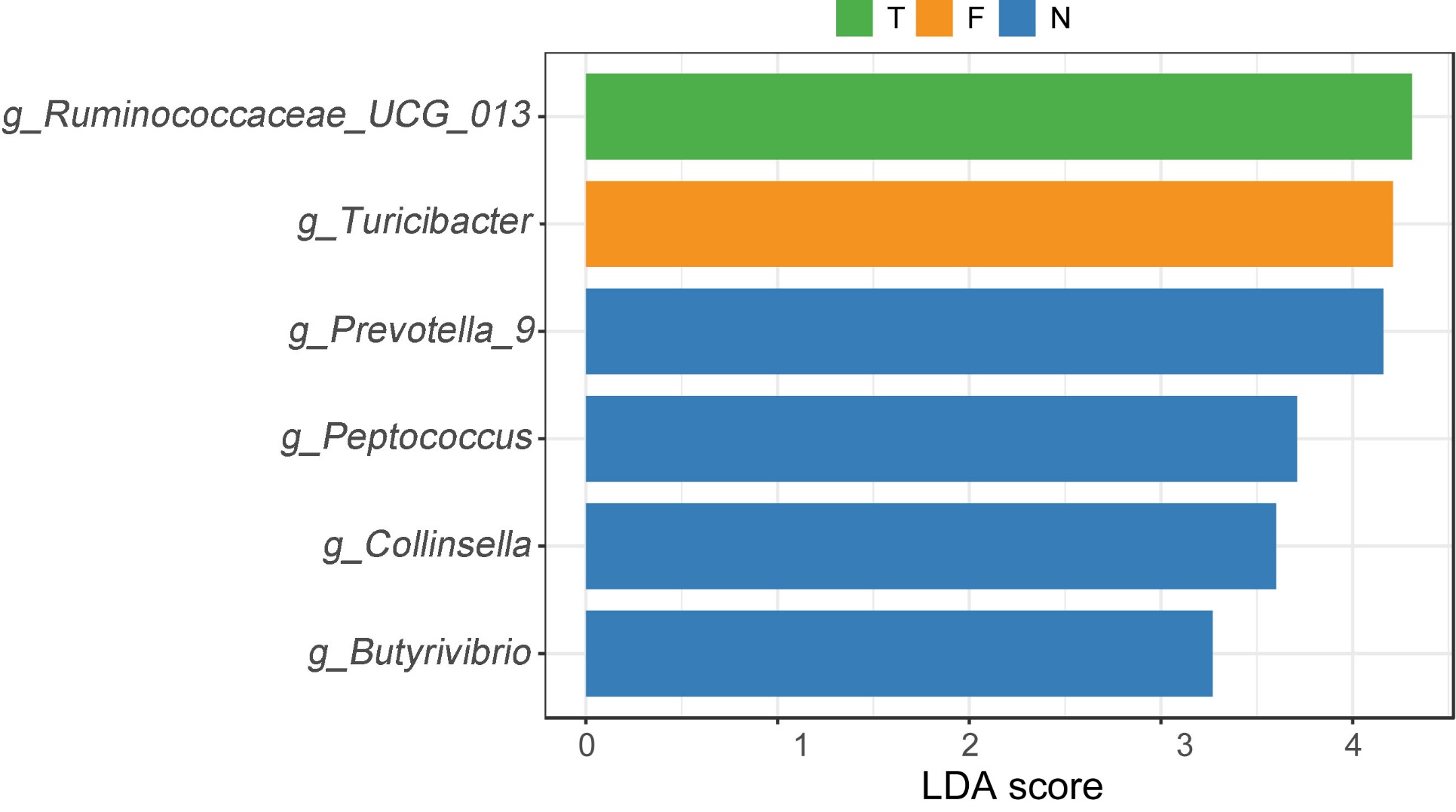


**Fig. S5.** Biomarkers identified at the genus level by LEfSe analysis of different ages


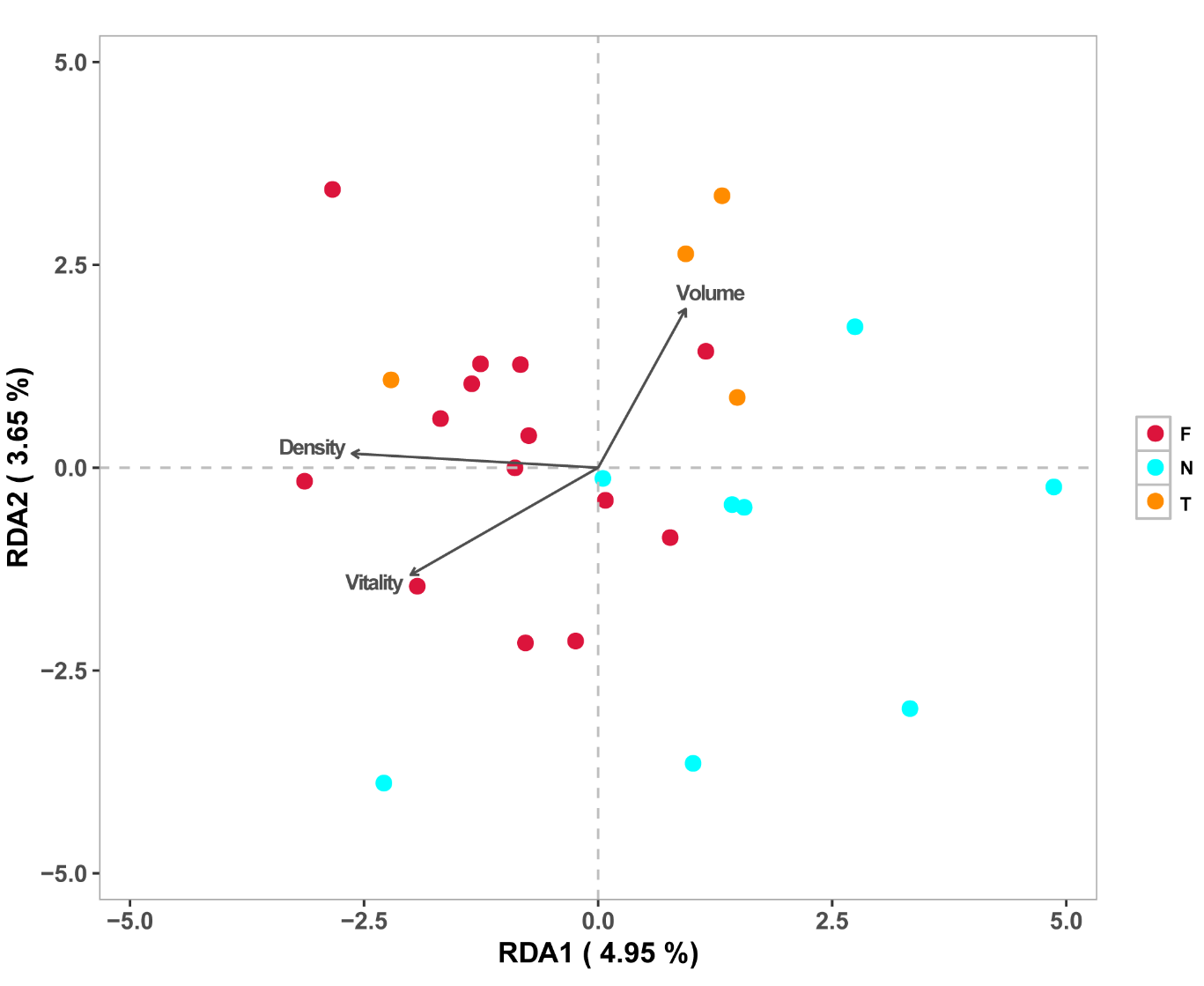


**Fig. S6.** Association analysis of different ages and environmental factors

**Table S1.** Semen quality of breeds and age

| Semen quality | Breeds | | | | Age (Months) | | | |
| --- | --- | --- | --- | --- | --- | --- | --- | --- |
|  | Duroc | Landrace | Yorkshire | P value | 9 | 15 | 20 | P value |
| Volume | 197.06±45.02 | 232.53±88.75 | 247.42±80.46 | 0.05433 | 205.97±72.73^b^ | 218.06±57.85^a^ | 282.7±100.65^a^ | 0.005849 |
| Density | 4.53±1.40^a^ | 3.88±1.23^b^ | 2.86±1.08^b^ | 2.95E-06 | 3.11±1.04^b^ | 4.15±1.24^a^ | 3.61±1.88^a^ | 0.001527 |
| Vitality | 0.8753±0.0754^b^ | 0.9136±0.0531^a^ | 0.8778±0.0527^b^ | 0.004105 | 0.8794±0.0495 | 0.8940±0.0737 | 0.8935±0.0500 | 0.1019 |

**Note:** The table shows the P-values of the overall differences between the groups as measured by the Kruskal-Wallis nonparametric test and the markers of the significance level of the differences as measured by the Dunn's Test after the fact.

**Table S2.** Predicted functional differences in the KEGG pathways.

| Class1 | Class2 | Relative frequency (mean$\pm$std) | | |
| --- | --- | --- | --- | --- |
|  |  | Duroc | Landrace | Yorkshire |
| Organismal Systems | Aging | 0.273$\pm$0.006 | 0.267±0.007 | 0.272$\pm$0.004 |
| Metabolism | Amino acid metabolism | 6.692$\pm$0.064 | 6.674$\pm$0.070 | 6.684$\pm$0.038 |
| Metabolism | Biosynthesis of other secondary metabolites | 1.058$\pm$0.024 ^a^ | 1.028$\pm$0.015 ^a,c^ | 1.059$\pm$0.027 ^c^ |
| Human Diseases | Cancers: Overview | 0.483$\pm$0.010 | 0.493$\pm$0.016 | 0.479$\pm$0.010 |
| Human Diseases | Cancers: Specific types | 0.054$\pm$0.008 | 0.055$\pm$0.004 | 0.056$\pm$0.002 |
| Metabolism | Carbohydrate metabolism | 9.277$\pm$0.144 | 9.315$\pm$0.183 | 9.183$\pm$0.156 |
| Human Diseases | Cardiovascular diseases | 0.002$\pm$0.001 | 0.002$\pm$0.001 | 0.002$\pm$0.000 |
| Cellular Processes | Cell growth and death | 0.562$\pm$0.016 | 0.549$\pm$0.008 ^c^ | 0.568$\pm$0.010 ^c^ |
| Cellular Processes | Cell motility | 1.155$\pm$0.107 | 1.157$\pm$0.206 | 1.333$\pm$0.212 |
| Cellular Processes | Cellular community - eukaryotes | 0.000$\pm$0.000 | 0.000$\pm$0.000 | 0.000$\pm$0.000 |
| Cellular Processes | Cellular community - prokaryotes | 1.449$\pm$0.033 | 1.452$\pm$0.024 | 1.450$\pm$0.037 |
| Organismal Systems | Circulatory system | 0.000$\pm$0.000 | 0.000$\pm$0.000 | 0.000$\pm$0.000 |
| Organismal Systems | Development | 0.000$\pm$0.000 | 0.000$\pm$0.000 | 0.000$\pm$0.000 |
| Organismal Systems | Digestive system | 0.054$\pm$0.015 | 0.051$\pm$0.019 | 0.047$\pm$0.006 |
| Human Diseases | Drug resistance: Antimicrobial | 0.915$\pm$0.015 | 0.916$\pm$0.025 | 0.910$\pm$0.013 |
| Human Diseases | Drug resistance: Antineoplastic | 0.003$\pm$0.001 | 0.004$\pm$0.001 | 0.003$\pm$0.001 |
| Human Diseases | Endocrine and metabolic diseases | 0.219$\pm$0.003 | 0.221$\pm$0.004 | 0.223$\pm$0.007 |
| Organismal Systems | Endocrine system | 0.604$\pm$0.019 | 0.587$\pm$0.012 | 0.606$\pm$0.023 |
| Metabolism | Energy metabolism | 3.974$\pm$0.096 | 3.953$\pm$0.051 | 3.965$\pm$0.082 |
| Organismal Systems | Environmental adaptation | 0.189$\pm$0.020 | 0.187$\pm$0.018 ^c^ | 0.203$\pm$0.013 ^c^ |
| Organismal Systems | Excretory system | 0.022$\pm$0.003 | 0.022$\pm$0.004 | 0.022$\pm$0.003 |
| Genetic Information Processing | Folding, sorting and degradation | 1.604$\pm$0.037 | 1.603$\pm$0.039 | 1.628$\pm$0.025 |
| Metabolism | Global and overview maps | 42.352$\pm$0.096 | 42.338$\pm$0.068 | 42.299$\pm$0.161 |
| Metabolism | Glycan biosynthesis and metabolism | 1.372$\pm$0.020 | 1.317$\pm$0.064 | 1.354$\pm$0.086 |
| Human Diseases | Immune diseases | 0.041$\pm$0.006 | 0.040$\pm$0.006 | 0.038$\pm$0.003 |
| Organismal Systems | Immune system | 0.085$\pm$0.011 | 0.083$\pm$0.010 ^c^ | 0.092$\pm$0.003 ^c^ |
| Human Diseases | Infectious diseases: Bacterial | 0.464$\pm$0.008 | 0.473$\pm$0.017 | 0.471$\pm$0.018 |
| Human Diseases | Infectious diseases: Parasitic | 0.032$\pm$0.008 | 0.032$\pm$0.007 | 0.027$\pm$0.004 |
| Human Diseases | Infectious diseases: Viral | 0.007$\pm$0.002 | 0.007$\pm$0.002 | 0.006$\pm$0.001 |
| Metabolism | Lipid metabolism | 1.831$\pm$0.015 ^b^ | 1.842$\pm$0.033 ^c^ | 1.810$\pm$0.018 ^b,c^ |
| Environmental Information Processing | Membrane transport | 3.665$\pm$0.241 | 3.716$\pm$0.151 | 3.595$\pm$0.147 |
| Metabolism | Metabolism of cofactors and vitamins | 4.128$\pm$0.042 | 4.129$\pm$0.037 | 4.133$\pm$0.097 |
| Metabolism | Metabolism of other amino acids | 1.241$\pm$0.015 ^b^ | 1.244$\pm$0.032 | 1.220$\pm$0.019 ^b^ |
| Metabolism | Metabolism of terpenoids and polyketides | 1.119$\pm$0.010 | 1.120$\pm$0.012 | 1.106$\pm$0.016 |
| Organismal Systems | Nervous system | 0.184$\pm$0.012 ^b^ | 0.189$\pm$0.010 | 0.195$\pm$0.002 ^b^ |
| Human Diseases | Neurodegenerative diseases | 0.110$\pm$0.005 | 0.109$\pm$0.008 | 0.106$\pm$0.003 |
| Metabolism | Nucleotide metabolism | 4.013$\pm$0.046 | 4.012$\pm$0.037 | 4.010$\pm$0.034 |
| Genetic Information Processing | Replication and repair | 3.281$\pm$0.027 | 3.282$\pm$0.015 | 3.293$\pm$0.032 |
| Organismal Systems | Sensory system | 0.000$\pm$0.000 | 0.000$\pm$0.000 | 0.000$\pm$0.000 |
| Environmental Information Processing | Signal transduction | 2.468$\pm$0.079 | 2.505$\pm$0.093 | 2.529$\pm$0.096 |
| Environmental Information Processing | Signaling molecules and interaction | 0.047$\pm$0.001 | 0.047$\pm$0.000 | 0.047$\pm$0.001 |
| Human Diseases | Substance dependence | 0.000$\pm$0.000 | 0.000$\pm$0.000 | 0.000$\pm$0.000 |
| Genetic Information Processing | Transcription | 0.182$\pm$0.007 | 0.184$\pm$0.004 | 0.184$\pm$0.007 |
| Genetic Information Processing | Translation | 3.903$\pm$0.037 | 3.912$\pm$0.033 | 3.939$\pm$0.050 |
| Cellular Processes | Transport and catabolism | 0.241$\pm$0.024 ^a^ | 0.210$\pm$0.015 ^a^ | 0.232$\pm$0.025 |
| Metabolism | Xenobiotics biodegradation and metabolism | 0.644$\pm$0.023 | 0.672$\pm$0.030 ^c^ | 0.621$\pm$0.025 ^c^ |

The same small-letter (a, b and c) indicates statistical significance between groups (p-value < 0.05), unmarked indicates no difference.
